# Ruminant livestock farmers and industry are leading innovation to deliver human nutrition and improved environmental outcomes through sector lifecycle collaboration: a review of case studies

**DOI:** 10.1093/af/vfae050

**Published:** 2025-04-05

**Authors:** Enrike Maree, James Blignaut, John Gilliland, Michael R F Lee, Pablo Manzano, Terry McCosker, Lindeque du Toit, Wayne Truter, Ben Weinheimer, Rod Polkinghorne

**Affiliations:** Department of Animal and Wildlife Sciences, University of Pretoria, Hatfield, South Africa; GOALSciences, Rapperswil, Switzerland; School of Public Leadership, Stellenbosch University, Stellenbosch, South Africa; ASSET Research, Sedgefield; South African Environmental Observation Network (SAEON), Pretoria, South Africa; Queens University, Belfast, Northern Ireland Harper Adams University, Sustainable Farm Networks (SFN),United Kingdom; Harper Adams University, Newport, Shropshire,United Kingdom; Basque Centre for Climate Change (BC3), Leioa, Spain; Ikerbasque — Basque Foundation of Science, Bilbao, Spain; Carbon Link Limited, Brisbane, Queensland, Australia; Department of Animal and Wildlife Sciences, University of Pretoria, Hatfield, South Africa; Resource Consulting Services Pty Ltd, Carbon Link, Yeppoon, Queensland, Australia; Green Futures Hub: Centre of Mineral Biogeochemistry, Faculty of Natural and Agricultural Sciences, University of the Free State, Bloemfontein, South Africa; FPLRI Research, Technology and Innovation Institute, Boschkop, South Africa; Texas Cattle Feeders Association (TCFA), Amarillo, Texas; Birkenwood Pty Ltd, Blandford, Australia

**Keywords:** adaptive grazing, carbon sequestration, emissions, grazing, intensive feeding

**Implications**

- Well-managed systems like adaptive grazing and silvopasture enhance soil health, biodiversity, and water retention while reducing greenhouse gas emissions.
- Intensive feeding practices, from supplementation to feedlots, increase meat production and manage emissions effectively through controlled feeding and manure strategies.
- Combining sustainable grazing with intensive systems balances land use, nutrition, and emissions reduction, addressing global food demand.
- Livestock’s up-cycling efficiency converts inedible grasses and by-products into nutrient-dense food, critical for food security.
- Farmers and industry leaders, through innovation and life-cycle analysis, use data-driven decisions to optimize sustainability, showcasing livestock’s essential role in achieving environmental and nutritional goals in agriculture.

## Introduction

Cattle farming has become a focal point in climate change discussions, as livestock agriculture reportedly accounts for 12% to 14.5% of global greenhouse gas emissions (Gerber et al., 2013; FAO, 2023). However, grazing ruminants, when managed properly, can potentially offset these emissions and create carbon sinks through effective carbon removal in soil and above-ground biomass –hereafter referred to as “flux fixation”—and ecological restoration (Mattila et al., 2021; Manzano et al., 2023). Additionally, there are several pathways to reduce the emissions profile of ruminant agriculture, which is essential for achieving zero hunger, improved nutrition, and sustainable agriculture (FAO, 2023). As such, delivering in concert with the Paris Climate agreement 1a—to hold the increase in global temperature to well below 2 °C above pre-industrial levels and pursue efforts to limit it to 1.5 °C, and 1b—to do this in such a way that does not threaten food production (United Nations, 2024).

These seemingly antagonistic goals can be symbiotic under careful, best-practice management. Carefully managed increased grazing-livestock production and efficiency align with improved soils, enhanced climatic resilience, increased biodiversity, and net carbon removal (Gibbons, 2020; Teague and Kreuter, 2020). Regenerative grazing is an agro-ecological approach that utilizes soil health and adaptive livestock management principles, rooted in the relationship between grasslands and ruminants, to transform modern agriculture by improving farm profitability, human and ecosystem health, and food system resilience. These systems can also up-cycle human inedible grasses, leaves, woody material, and agricultural by-products into high-quality human protein, delivering important ecosystem services simultaneously (Gibbons, 2020). Such systems optimize resource use, reduce waste, and enhance nutrient cycles, which are crucial for supporting a growing global population, contributing to a circular economy, and optimizing land use for sustainable food production (Thompson et al., 2023; Van Loon, 2023).

The role of livestock is further underscored by the commercial and technical challenges in synthesizing alternative protein products as ingredients (Tubb and Seba, 2021; Wood et al., 2023). The natural evolution of ruminants over many millions of years and the subsequent domestication of livestock has resulted in a highly efficient and environmentally beneficial system. It is crucial to acknowledge that properly managed livestock farming can play a key role in feeding a growing population while promoting environmental sustainability (Beal et al., 2023).

However, the diversity of managed grazing systems indicates that a one-size-fits-all approach may require reconsideration by adapting the relevant systems to the geographic, socioeconomic, and environmental conditions in which they occur (Asner et al., 2004; Mattila 64 et al., 2021; Copeland et al., 2023; Manzano et al, 2025). Based on these considerations, this article provides an overview of various managed grazing systems and their integration into land-use practices, considering sustainability indicators such as land use, carbon flux fixation, soil health, productivity, and socioeconomics. By examining both unpublished case studies and published data from commercial innovation leaders in the livestock sector, it highlights the potential of managed grazing, with varying intensities of supplementary feeding, in supporting sustainable agricultural practices and addressing climate change and malnutrition.

## Global Context

**Figure 1.**
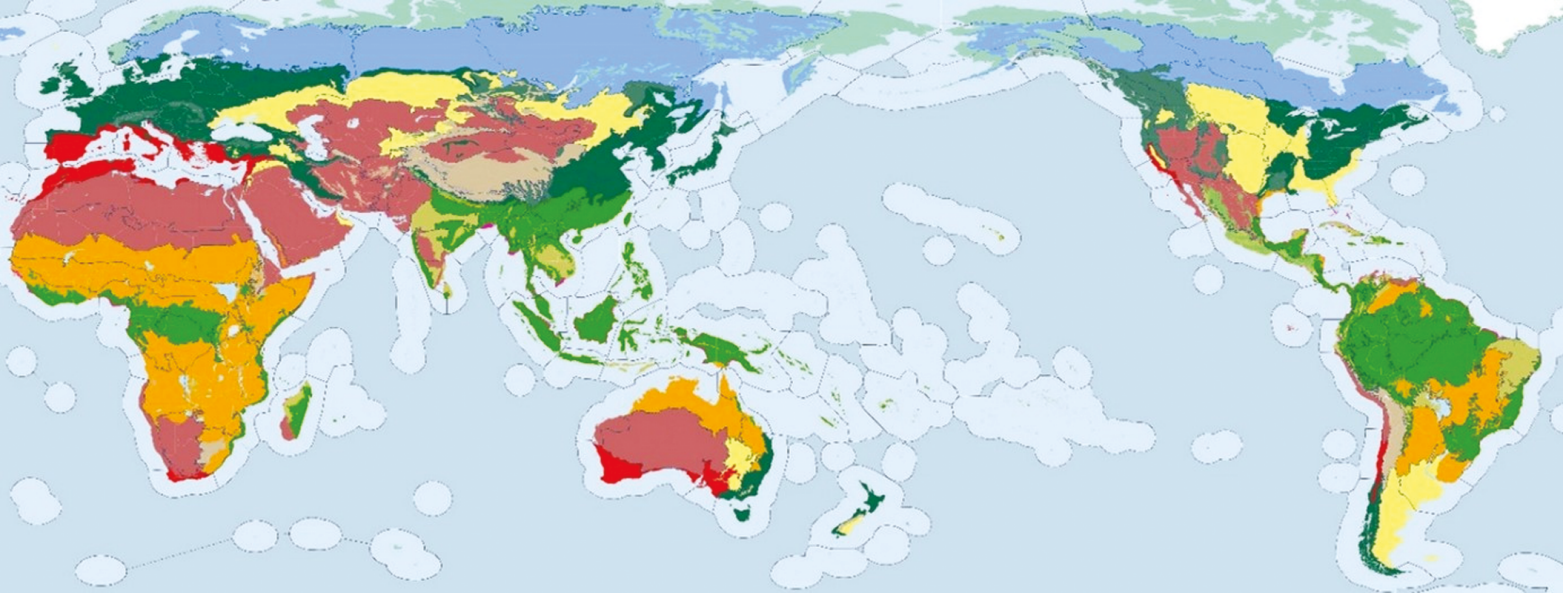
The Earth’s 14 major biomes (including six subtypes of the generic forest biome (Burkart, 2023). Deserts & Xeric Shrublands (rust); Mountain Grasslands & Shrublands (beige); Temperate Grasslands, Savannas & Shrublands (yellow); Tropical & Subtropical Grasslands, Savannas & Shrublands (orange); Flooded Grasslands & Savannas (light blue); Mangroves (pink); Mediterranean Forests, Woodlands & Scrub (red); Temperate Broadleaf & Mixed Forests (dark green); Temperate Conifer Forests (gray-green); Tropical & Subtropical Coniferous Forests (light green); Tropical & Subtropical Dry Broadleaf Forests (olive green); Tropical & Subtropical Moist Broadleaf Forests (bright green); Boreal Forests/Taiga (medium blue); Tundra (teal).

Globally, various ecoregions exhibited in Figure 1, are characterized by unique climatic conditions, vegetation types, and land use practices (Sulc and Franzluebbers, 2014; Smith et al., 2018). For instance, deserts and xeric shrublands, covering 19.8 million km² (out of 130 million total ice-free land), experience low rainfall and high temperatures, making them suitable primarily for nomadic grazing and wildlife habitats, with limited potential for cropping (Asner et al., 2004; Burkart, 2023). In contrast, temperate grasslands, savannas, and shrublands, spanning 9 million km², have moderate temperatures and seasonal rainfall, making them suitable for crop production and livestock grazing.

**Figure 2.**
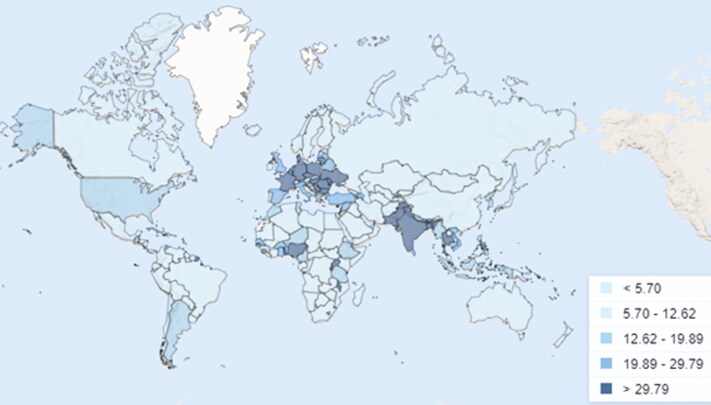
Global arable land, as a percentage of available land (World Bank, 2021).

**Figure 3.**
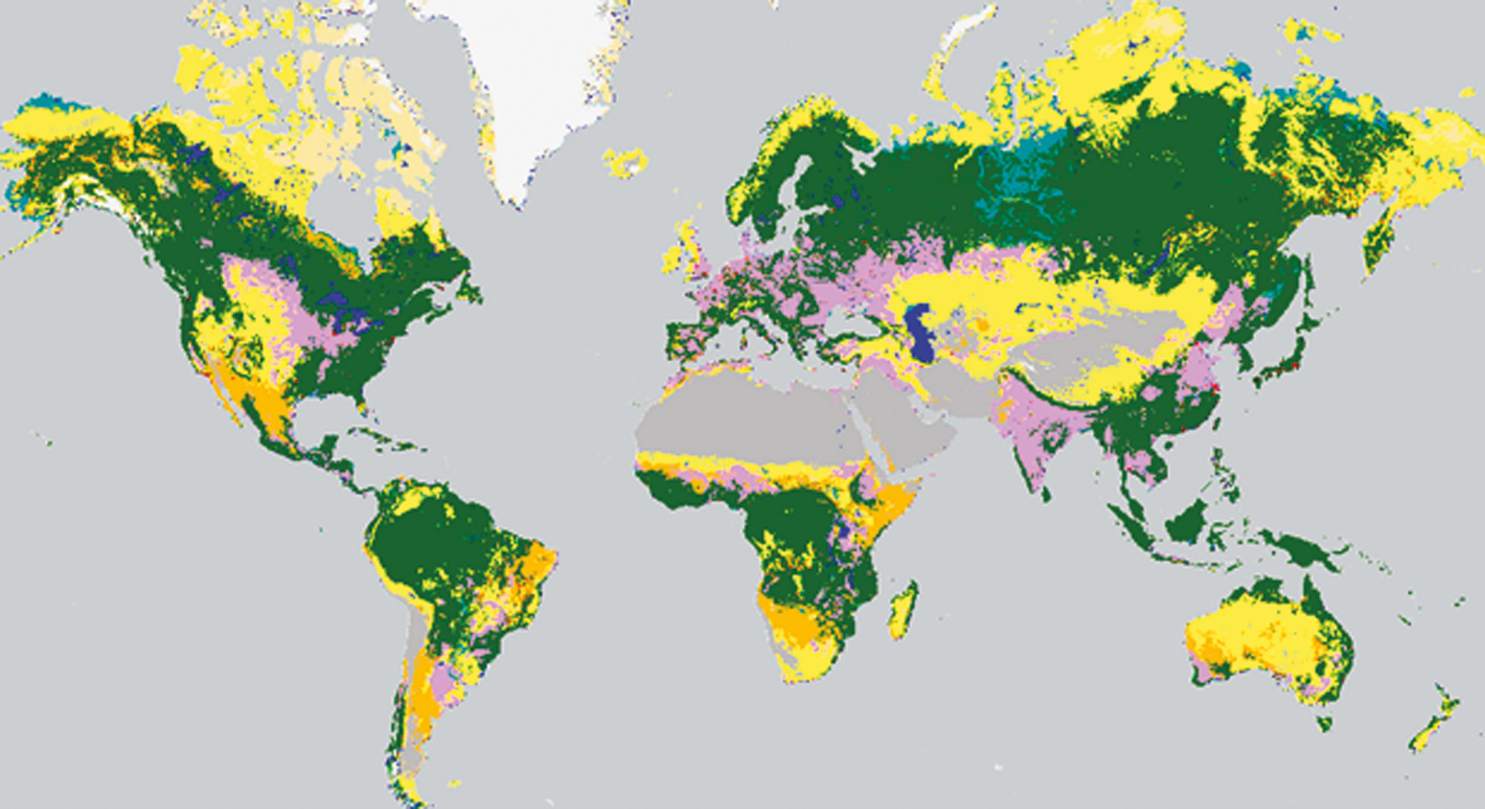
Global land use map (Copernicus, 2019). Blue: water bodies; white: snow; red: built-up; pink: cropland; gray: sparse vegetation/bare; beige: moss & lichen; turquoise: herbaceous wetland; yellow: herbaceous vegetation; orange: shrubland; green: forests.

These distinctions become more apparent when examining the percentage of arable land per country in Figure 2 and the corresponding areas currently used for cropland in Figure 3, owed to the unique geographic and climatic conditions (World Bank, 2021; Wu et al., 2018). Maximizing cropland production requires optimizing efficiency in the limited zones of available arable land (World Bank, 2018; Wu et al., 2018). Similarly, non-arable lands characterized by forests and shrublands require innovative strategies to make them productive while maintaining ecological stability. However, it is insufficient for each country to only optimize itself, since increasing portions of the global population have no prospect of food self-sufficiency, even with optimal technological advancements and production (Schramski et al., 2019). For instance, city–states such as Singapore or UAE, or countries with expected high population growth such as Egypt or Nigeria, will not be capable of being self-sufficient, thus a global effort is required to optimize all land-use types for the distribution of products globally. Since between 20% and 60% of the input biomass for human food production becomes non-edible by-products that can be consumed by livestock, grazing systems can contribute to this effort by up-cycling underutilized biomass or non-arable areas into valuable food resources while enhancing soil health and biodiversity through manure deposition and grazing activity (Herrero et al., 2013; van Selm et al., 2022; Thompson et al., 2023). Van Hal (2020) suggests that livestock can provide an average of 7-30g of human digestible protein per capita per day by up-cycling low-quality feed (such as grassland, by-products from food production, crop residues, etc.).

## An Overview of Grazing Systems

Grazing systems are globally grouped by a variety of definitions such as rotational, selective, holistic, or adaptive; versus selective, continuous grazing, or similar derivatives (Hawkins, 2017; Mann and Sherren, 2018). For the purposes of this article, grazing systems have been distinguished into adaptive grazing and continuous grazing. Adaptive grazing, occasionally known as rotational grazing, involves adjusting the grazing regime daily based on animal requirements, seasons, and pasture conditions. Continuous grazing, by contrast, allows livestock to graze selectively in large paddocks and generally offers fewer ecological benefits compared to adaptive systems (Harmel et al., 2021). Continuous grazing could also apply rotational practices in a large paddock or camp system, and, conversely, rotational grazing with too long or too short periods of rest (non-grazing) could also be detrimental to veld and pasture health (Asner et al., 2004; Sammuelson and Rood, 2004). Continuous grazing can prevent biomass buildup, reduce the risk of fires, improve herd fertility, and lead to acceptable livestock body condition scores while requiring lower fencing and managerial inputs (Orr et al, 2019; Harmel et al., 2021). Nonetheless, ecological benefits are predominantly observed with adaptive grazing systems, where forages are allowed time to recover by regrowing during times of non-grazing, and soil health indicators are improved (Hawkins, 2017; Harmel et al., 2021).

**Figure 4.**
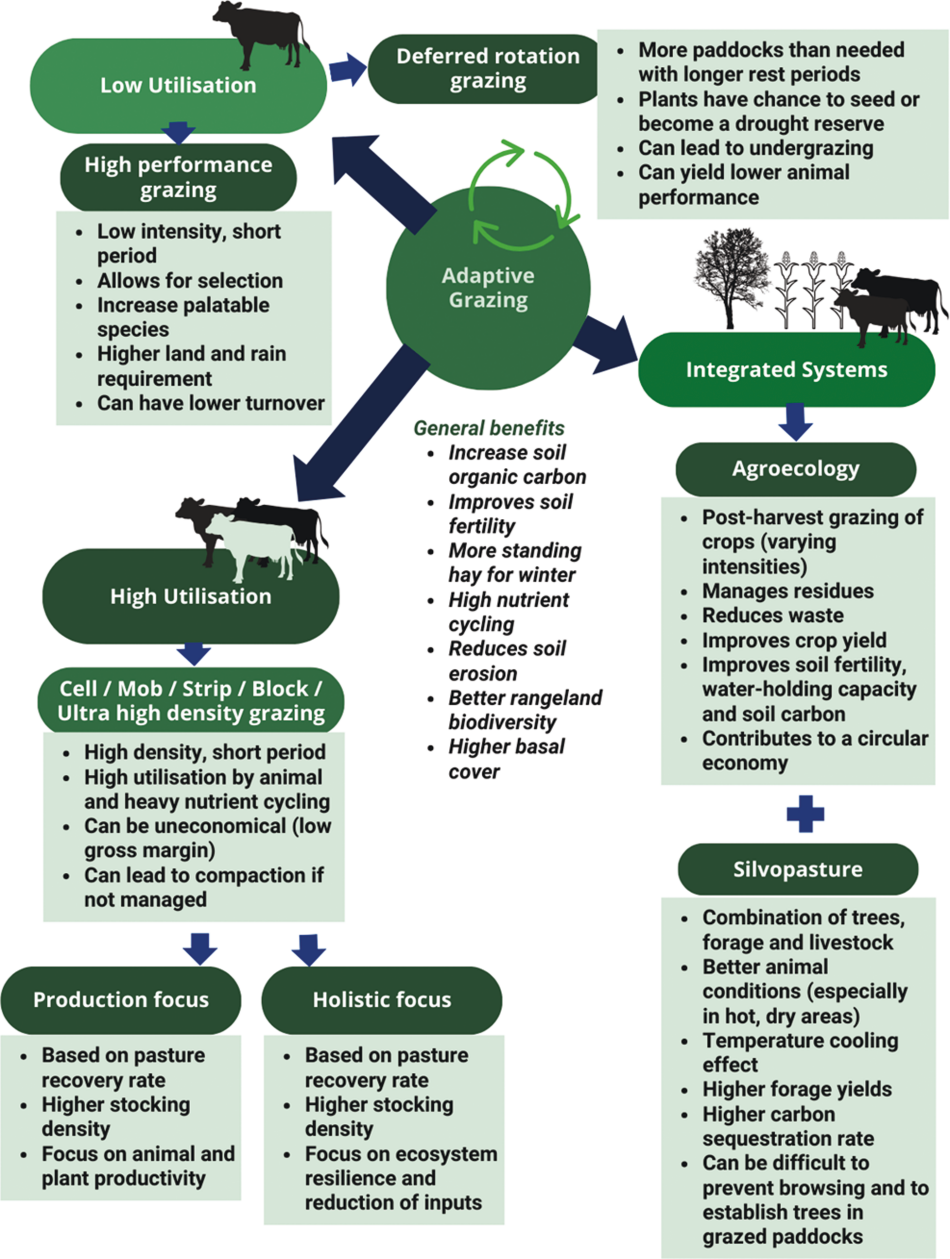
Adaptive grazing systems. Adapted from: McCosker, 2000; Fornara et al., 2011; Hawkins, 2017; Jose and Dollinger; 2019;. Ritchie, 2020; Tham et al., 2020; Harmel et al., 2021; Smith et al., 2021; Zeppetello et al., 2022; Thompson et al., 2023; Brewer et al., 2023.

Adaptive grazing (AG) systems, such as high-density grazing, strip grazing, holistic/mob grazing, cell grazing, and grazing systems integrated into silvopastoral and agroforestry systems—summarized in Figure 4—enhance soil carbon flux fixation, improve soil fertility, and increase water-holding capacity, contributing to rangeland restoration (Spackman and Allison, 2023; Thompson et al., 2023). In general, these systems allow for better pasture utilization, higher plant and animal productivity, as well as better nutrient cycling, soil health, and over-all ecosystem health. Silvopasture and agro-ecological systems diversify income, support livelihoods, and reduce temperatures, creating favorable conditions for livestock. Moreover, anecdotal evidence accumulates in all corners of the world, that these systems contribute to carbon sequestration and improved farm productivity (Chen et al., 2021).

Although multiple benefits are associated with managed grazing, the type of grazing system may be dependent on the environment in which it is applied. For example, a meta-analysis comparing high-density grazing with continuous grazing found no significant differences in overall plant basal cover and biomass, yet in higher precipitation areas it showed positive effects on plant basal cover (Hawkins, 2017). Similarly, silvopasture systems have found better forage response with C3 (or cool-climate grasses) in comparison to C4 (or warm-climate grasses) (Jose and Dollinger, 2019). This suggests that the benefits of grazing may vary based on local environmental conditions, and germplasm and require precise management to avoid overgrazing or rangeland degradation.

On the other hand, zero-grazing systems- while not an active grazing strategy, may be necessary during certain periods for degraded rangelands to recover sufficiently to maintain livestock or wildlife (Samuelson and Rood, 2004). Temporarily excluding livestock from a degraded rangeland or previously overgrazed rangeland, allows natural vegetation succession and the successful establishment of, for example, riparian cottonwoods (Samuelson and Rood, 2004; Bezner Kerr et al., 2021). However, this requires management and timeous cessation to prevent declines in soil and plant diversity due to long-term use (Schrama et al., 2023). In this context, unpublished and published case studies have been reviewed in section 4 to further assess the effects of each grazing strategy in various conditions on the ecosystem in which they were implemented.

## Case Studies: Livestock Systems, Productivity, and Ecosystem Impacts

The following case studies from innovation-leading livestock farmers and the wider industry, both published and unpublished, illustrate the diverse ecosystem responses associated with different grazing and feeding systems. The case studies presented from Northern Ireland and Australia, summarized in Table 1 and reported in supplementary Material (S1 and S2) represent diverse environmental contrasts while sharing positive outcomes from specific regional management. Further evidence, with a focus on incorporating multiple vegetative species in the production system, is summarized in Table 1, of which some are elaborated on in the supplementary material (S1 to S4).

**Table 1.** Grazing systems and their associated responses

Area	System	Responses
New Zealand^1^ A) Waikato; B) Canterbury	Adaptive grazing (dairy)	NECB of 12 ± 30 g C m² y⁻¹, NEP:283 ± 31 g C m² y⁻¹ Increased diversity led to carbon neutrality or carbon sources
	Crop > pasture transitions	Net carbon loss (higher in longer phases) Larger C losses in allophonic, gley, and organic soils
	Supplemental feed crops	Reduced NECB to -32 ± 41 g C m² y⁻¹.
USA^2^ -Texas	Adaptive grazing	Soil respiration changed from 35.3 to 64.6 mg CO_2_/kg soil and WEOC from 187.2 to 232.2 mg/kg. Higher forage production (+1,500 kg/ha) Lower cost ($37,050) and revenue ($38,548) Higher standing forage 20% higher daily liver weight gain
	Continuous grazing	Higher winter crude protein and digestible organic matter Higher cost ($48,971) and revenue ($42,897)
United Kingdom A) Various^3^ B) Rothamstead research centre^4^	Zero grazing	Declines in diversity for soil organisms and plants. No difference in bacterial diversity. Nematode, mite, and springtail diversity increased by 15%, 5%, and 15%, respectively.
	Adaptive grazing	SOC increased by 1.24 t C/ha/year. Higher pasture growth (39%–54%), better sward composition and animal production Reduced greenhouse gas emissions Liveweight of 483–890 kg LW/ha
	Continuous grazing	Decrease of soil carbon by 0.45 t C/ha/year Liveweight of 367–585 kg LW/ha Less efficient carbon sequestration and higher impacts
Northern Ireland, Londondery^5^	Grassland transition to woodland/silvopasture	+ SOC by 11% and 47% (depending on the depth of measure of 0–15cm or 15–30cm)
	Woodland transition to grassland	Decreased SOC
Ireland A) Dowth research farm^6^ B) Loughhall^7^	A) Adaptive grazing (multispecies swards and woodlands) B) Silvopasture (compared with traditional grasslands)	A) Nitrogen use was reduced by 65%, ADG improved by 20%, increased earthworm population by 300%. The water infiltration rate improved 14-fold. GHG emissions per kg of meat were reduced by 26%, with a 53% reduction in wheat production emissions. Some farms achieved net zero 97% of total carbon was stored in soil. B) Improved tree growth, increased soil carbon, extended grazing season of 17 weeks, improved soil infiltration vs regular grasslands. Farm emissions were offset by 3.3%. 77.28 t C/ha stored over 21 years.
USA, California^8^	Crop-livestock systems	No differences in physical soil properties Higher microbial, bacterial, fungal and actinomycete content in soil Higher microbial biomass carbon at depths of 0–15 cm, 15–30 cm, and 3–45 cm, decreasing in effect per layer. Increased soil organic carbon at 15–30 cm and 30–45 cm (+ 3.5 g/ and + 2.1 g/kg)
Sout Africa, North-East Free State^9^	Adaptive grazing	Decreaser grasses increased by 1.2%, indicating improvement in veld condition. Increaser grasses decreased by 1.8%, showing reduced disturbance and overgrazing effects. VCS increased by 7.9%. Biomass production is 5212 kg/ha. Grazing capacity is 2.7 ha/LSU. Grass species diversity increased by six species, indicating better biodiversity.
	Continuous grazing	Presence of decreaser grasses at 15%. Increaser grasses decreased by 38.7%, still high due to selective grazing management. VCS decreased by 10%. Biomass production is moderate at 3,153 kg/ha. Grazing capacity 5 ha/LSU. Grass species diversity remained constant at 11 species, indicating stable but lower biodiversity.
	No grazing	Decreaser grasses decreased by 13.2 = a complete loss of palatable species. Increaser grasses increased by 20.1%, indicating undergrazing and accumulation of unpalatable species. VCS decreased by 6.3%, Biomass production is 6760 kg/ha but includes a high proportion of moribund material. Grazing capacity is 3 ha/LSU, likely overestimated due to high volume of low-quality biomass. Grass species diversity decreased by 8 species, indicating a loss of biodiversity.

NECB, net ecosystem carbon balance; NEP, Net ecosystem production; SOC, Soil organic carbon; VCS, veld condition score; Sources: 1) Wall et al, 2024; 2) Harmel et al., 2021; 3) Schrama et al., 2023; 4) Rivero et al., 2024; 5) Buffaro and Gilliland, 2023; 6) Gilliland, 2023; 7) McAdam, 2016; 8) Brewer et al, 2023; 9) Van Oudtshoorn, 2021.

The abovementioned case-studies show that if managed well, integrative approaches to managing grazing livestock—aimed at enhancing ecosystem biodiversity and resilience—can yield overall positive benefits in economic production, efficiency, and ecosystem health. Much can be learned from these case studies to improve existing grazing systems on a global scale and to ensure that ruminant production becomes more sustainable. However, considerations such as soil type, previous land conditions, and geoclimatic limitations must be taken into account before scaling these principles. In many cases, for example, grazing systems integrate with cropping systems, planted pastures, or supplementary feeding—particularly in drought or frost-sensitive areas where hot, dry summers or winters below freezing temperatures or excess rainfall may influence available forage. Livestock conditions, or soil conditions, necessitating supplementation or requiring cut-and-carry rather than on-site grazing (Godde et al., 2019; Bogale and Erena, 2022).

The critical role of complementary feeding systems must also be recognized in the quest for optimum livestock, human nutrition, and environmental outcomes. These enhance grazing via four key mechanisms: (1) enabling maximum utilization of pasture; (2) efficient conversion of human inedible by-products; (3) return of manure to crop or pasture; and (4) avoiding emissions through increased growth rates and lower age at slaughter.

High grazing-system efficiency relies on expert management of livestock numbers related to available feed, with this subject to extensive seasonal variation. To maximize efficiency and related soil and environmental benefits without damage through overgrazing or excessive compaction, livestock are transferred to a barn or pen area either seasonally or for the ensuing period to harvest. These systems may utilize forage conserved during seasonal peaks and/or locally available by-products or non-human-edible feed materials, such as seen in Table 2 (Eisler et al, 2014). This utilization and return of manure and effluent to crops or pasture provides direct environmental benefit and indirect through a reduction in synthetic fertilizer application. Annual slurry applications from livestock housing have shown continual soil benefit over periods of 43 years (Fornara et al., 2016). In California, dairy lagoons significantly reduce methane emissions by capturing and processing manure (Parker et al., 2017). Although higher numbers of dairy lagoons are associated with higher initial methane emissions, covered lagoons prevent methane escape and reduce methane emissions from dairy systems by up to 100% in the case of anaerobic digestion adoption (Greene et al., 2024). Some facilities also convert it into renewable natural gas, lowering greenhouse gas emissions and supporting the state’s carbon reduction goals by becoming a carbon-sink in this manner. (Leytem et al., 2017; Parker et al., 2017). In addition, a study by Lisboa and Lansing (2013) demonstrated that co-digestion of farm or food processing wastes with dairy manure can significantly enhance biofuel-methane production, with increases ranging from 67% to 2,940%, depending on the type of waste used, increasing system efficiency.

**Table 2.** Feedlot capacity, turnoff and commodity data in 5 surveyed Australian feedlots 2023

Feedlot		A			B			C			D			E		
Capacity (Head)			30,000			3,000			17,000			22,500			20,000	
Annual turnoff (Head)			90,000			8,000 to 10,000			51,000			60,000			60,000	
% Domestic (< 100 days on feed)			0%			90%			0%			35%			0%	
% Short fed export (< 100–200 days on feed)			100%			10%			90%			35%			100%	
% Long fed export (> 200 days on feed)			0%			0%			10%			30%			0%	
Distance to principal abattoir(s) (km)			350/400			160			100			80			270/480	
**Ration commodities.**		**Tonne**	**Grade**	**Km Purchase Rdius (km)**	**Tonne**	**Grade**	**Km Purchase Rdius (km)**	**Tonne**	**Grade**	**Km Purchase Rdius (km)**	**Tonne**	**Grade**	**Km Purchase Rdius (km)**	**Tonne**	**Grade**	**Km Purchase Rdius (km)**
**Grains**	Wheat	16,600	AUH2	350	1000	SFW1	60				60,000	SFW1	10 to 600			
		66,400	FED1	350										28,000	FED1	300
	Barley	6,500	Barley1	350	1,200	Barley1	10	62,000	Barley1	400	50,000	Barley1	10 to 600	28,000	Barley1	300
	Maize				2,500	Prime	40									
					2,500	Feed No 1	40									
**Pulses/Legumes**																
	Faba Beans										4,000	No 1	50 to 300			
**By-Products**	Almond hulls										3,600		600 to 800			
	Bread waste				4,500		125							6,000		250
	Dough				160		125									
	Oilseed meals							150		50				150		250
	Whole cottonseed	15,600		250	1,000		40	8,400		250	7,000		200	6,500		375
**Roughage**	Cereal hay	1,000		250							7000		10	2400		150
	Cereal straw	3,400		250	450		0				3500		50 to 700			
	Rice hulls															
	Lucerne hay				350		0	1100		250						
	Corn silage										13,000		0			
	Barley silage							35,000		50				1500		50
	Sorhum silage	20,000		50	2500		0									
**Liquids**	Oil	2,100		500				1400		500	1000		300 to 700	1700		250
	Molasses	2,000	CSBP-1	500							2500		300 to 700	400	CSBP-1	160
	Molasses based liquid supplement	4,300		500				2,500		600				2,500		160

In this context, intensive feeding practices, such as those implemented in feedlots and demonstrated in Supplementary Material (S3), are essential for cost-efficient meat production, allowing for increased production efficiency and shorter finishing periods –permitted manure management is implemented such as mentioned above. Feedlots are used globally, with millions of cattle managed under these systems. For instance, in the United States alone, there are over 26,000 feedlots, emphasizing their widespread adoption in modern meat production. While carbon flux fixation remains an important strategy for mitigating the impacts of food production, reducing direct emissions from livestock is equally critical—to which feedlots contribute through improved resource utilization and increased production efficiency.

Intensive feeding can provide necessary nutritional supplements during resource-scarce times, especially since stressed animals have been shown to emit more methane (Wolf et al., 2021; Borgale and Erena, 2022). Managed feeding programs that control intake have also proven to reduce emissions, further lowering the environmental footprint of livestock production (Méo-Filho et al., 2020; Galyean and Hales, 2023; Galloway et al., 2024). Intensive feeding programmes often utilize metabolic modifiers, which can reduce emissions and enhance cattle efficiency (Cooprider et al., 2011). Supplementation of feed additives, feeding grains, and managed feeding programs show that feedlots can reduce livestock emissions by 12%–40% while reducing the finishing time for cattle (Wiedemann et al., 2017; Ridoutt et al., 2022). As a result, current United States, intensive feeding systems only contribute about 14% of the total life cycle greenhouse gas emissions of beef production (Rotz, 2023).

However, effective manure management is crucial, as poor manure management can negate positive reduction effects by contributing to emissions (du Toit et al., 2013). Manual manure application, while not providing all the benefits of grazing, still yields multiple advantages, reducing cropping costs due to lower fertilizer inputs and optimal timing in the application thereof on crops (Brummerloh and Kuka, 2023). The Australian feedlot case study in Supplementary Material (S3) demonstrates this with the effective utilization of manure as also presented in Table 3. Nutrient circularity, where cattle consume non-edible grain by-products in intensive feeding systems, is another critical component. By utilizing discarded crops or by-products, intensive feeding systems reduce inputs and greenhouse gas emissions associated with livestock production (Kleinpeter et al., 2023).

**Table 3.** Estimated production and utilization of manure in five surveyed Australian feedlots 2023

Feedlot		A	B	C	D	E
Capacity (Head)		30,000	3,000	17,000	22,500	20,000
Annual turnoff (Head)		90,000	8,000 to 10,000	51,000	60,000	60,000
% Domestic (<100 days on feed)		0%	90%	0%	35%	0%
% Short fed export (<100–200 days on feed)		100%	10%	90%	35%	100%
% Long fed export (>200 days on feed)		0%	0%	10%	30%	0%
Distance to principal abattoir(s) (km)		350/400	160	100	80	270/480
**Total standard cattle units (SCU)**						
Domestic (<100 days on feed) 0.87 SCU			7047		18,270	
Short fed (<100–200 days on feed) 0.93 SCU		83,700	837	42,687	19,530	55,800
Long fed (>200 days on feed) 1.00 SCU				5,100	18,000	
	**Total SCU**	**83,700**	**7884**	**47,787**	**55,800**	**55,800**
**Liquid effluent disposal**						
- Evaporative ponds with prior solids sedimentation & drying		100%		100%		100%
- Spray application to adjacent company cropland			100%		100%	
**Solid manure treatment and utilization**						
- Windrowed & turned—Sold to local farmers		100%				
- Windrowed & turned—Sold to orchards & vineyards						28%
- Windrowed & turned—Company cropland			100%			
- Composted and screened—External sales farms & garden supplies						
	- Company cropland			50%	30%	72%
	- External sales local farm & garden supplies				70%	
	- Sold to city green waste plant			50%		
**Est Tonne manure solids (500 kg/SCU)**		**41,850**	**3942**	**23,894**	**27,900**	**27,900**
	- Est Tonne N (2.18% db)	912	86	521	608	608
	- Est Tonne P (0.80% db)	335	32	191	223	223
	- Est Tonne K (1.86% db)	778	73	444	519	519

The case studies presented illustrate the critical need for bespoke local management adaptation and innovation. Complementary supplemental feeding can counter normal or extreme seasonal variation challenges to grazing systems, which form the principal production base for the breeding herd and a considerable portion of growth to harvest. This complementarity is essential for meeting global human nutrition needs while ensuring environmental improvement. However, country-specific goals and limitations must be taken into consideration when evaluating intensive and non-intensive feeding practices. The case studies in the supplementary data illustrate that to manage these complex system interdependencies, it is necessary to capture data extensively and make them part of evidence-based managerial decision-making. De Lange et al (2025) in this issue, illustrate an example of how such production-system oriented evaluation systems could look like.

## Additional Considerations

### Carbon flux fixation is a welcome and important by-product of optimally managed 235 rangeland, but should not be the only optimization parameter

Carbon flux fixation in soils, as observed in the case studies mentioned, is a recognized benefit of grazing systems. In cropping systems, cropland typically acts as a net emitter of carbon rather than a carbon sink, with the lowest emissions observed in no-till continuous cropping systems (Sainju and Allen, 2024). When combined with grazing strategies, carbon can be returned to the soil, optimizing cropland production and yield while creating a carbon sink (Wall et al., 2024). However, a risk emerges if the sole focus is placed on the potential benefits that grazing can have regarding carbon flux fixation (Godde et al., 2020). For example, converting cropland to pasture can result in a carbon cost due to disturbance of the ecosystem, especially in well-managed ecosystems with high soil organic carbon (SOC) content (Blackwell et al., 2024; Wall et al., 2024).

Soil carbon, if not mineralized (sequestration—permanent locking up of carbon), however, is not stored permanently. Turn-over of soil organic matter occurs continuously over a range of timescales and is sensitive to management and climate factors, resulting in some soils being a net source or net sink of organic carbon. The challenge is to identify soils that have been depleted of carbon by farming practices (for example, intensively cultivated arable mineral and organic soils) or natural events that will be responsive to restoration by management that fosters soil carbon repletion such as the return of animal manures or grassland rotations. There are undoubtedly some circumstances in which carbon sequestration can be used to increase soil carbon storage, especially in depleted arable soils, that have a high potential to store more carbon (Janzen et al., 2022). Permanent grassland soils, however, will approach an equilibrium state as they age in which the quantity of carbon gained is equal to carbon losses. However, the period to reach this equilibrium will depend on many soil and management factors. As many grassland soils are relatively rich in organic carbon when compared to those elsewhere there may be challenges but also opportunities to manage these soils associated with maintaining or increasing existing SOC stocks. For grasslands that may have reached equilibrium grazing management will play a vital role in maintaining these carbon stocks as the main terrestrial carbon store. Another important factor as demonstrated in the case studies is that changes in grazing practice can shift that equilibrium i.e., there is still turnover of carbon within the soil, but if more organic matter is returned than degraded over a set period (e.g., grazing cycle) then there will be a net gain in carbon.

The effectiveness of carbon flux fixation through grazing is further mostly relevant in depleted soils and low rainfall zones as measured in the McCosker Australian case study where carbon drawdown of CO 2eq in a 5-year period over four properties was 61, 63, 53, and 32 tonne per tonne of livestock carried. In ecosystems with already high SOC content, the benefits of additional carbon sequestration may be smaller (Sarkar et al., 2020) although it should be noted that carbon may be stored beyond the depth analyzed in many studies. Additionally, the soil profile such as soil type or soil pH further influences the efficacy and potential for carbon sequestration (Mattila et al., 2021). The goal of the production system may further influence the results seen. Some high-density grazing strategies aimed at maximum livestock conditions often prioritize high dry-matter productivity on pasture, thus choosing plants that have a higher shoot-to-root ratio which are less effective at storing carbon (Wall et al., 2024). Similarly, poorly managed high-density grazing aimed at fixating carbon can lead to greater losses in body condition score or cattle numbers during winter without adequate standing hay or supplementary feeding.

Thus, the rate and maximum potential of carbon flux fixation in the production system, as well as the goal of the production system are limiting factors of using carbon flux fixation as the only indicator of climate mitigation. As is, reports state that based on current SOC stocks, current grazing systems need to improve their carbon flux fixation potential by 25% to 2,000% to be viable as standalone mitigation strategies (Wang et al., 2023). Since carbon flux fixation potential can be seasonally dependent and achieve a peak, after which it reaches equilibrium, focusing solely on carbon flux fixation may in turn lead to the use of inappropriate grazing strategies, potentially damaging species diversity and ecosystem resilience. As an example, one case study showed that although diverse pastures increased soil carbon flux fixation rates, some irrigated single specie pastures had higher carbon stocks (Wall et al., 2024). This underscores the importance of considering various unique results in each scenario are possible, and other important ecosystem benefits such as resilience can exist alongside carbon flux fixation.

Strategies such as silvopasture are another example, as it not only facilitates carbon capture above and below ground but also provides shelter belts that assist in climate control for livestock and consequent improved productivity (Zeppetello et al., 2022). Notwithstanding soil as a carbon capture approach, increasing soil carbon storage improves overall soil health (biological functioning) and water holding capacity through improved physical microscale structure. Grassland soil through its higher SOC has improved physical structure (more numerous and more connected soil pores) which increases both water storage and hydraulic conductivity (the ability of water and gases to move through the soil). Limiting organic carbon inputs and tillage degrade this structure and the hydraulic conductivity and water holding capacity are reduced as a consequence (Neal et al., 2020). This structure is important because it allows oxygen to move through the soil, limiting the volume of anoxic space (with low oxygen concentrations). Low oxygen forces shift microbial metabolism and result in nutrient losses from soils, particularly of nitrogen as nitrous oxide. In high carbon, well-structured and more oxygenated soils such as grasslands, microbes assimilate nutrients into biomass more effectively and nutrients are therefore retained in soil rather than lost. This response to organic carbon is observed in clay-loam soils, but not sandy-loam soils and this needs to be considered in on-farm and national management of soil carbon. From a practical perspective, this work demonstrates that organic carbon is an essential component of productive soils which is directly related to yield resilience by increasing the holding capacity and delivery of water to grass and crops, and storage of nutrients in soil by increasing assimilation in soil biomass. The increased water-holding capacity of high-carbon soils also has practical implications for reducing flood risks, a vital service of our grassland soils. Therefore, farmers and policymakers should adopt a holistic approach to environmental sustainability, focusing on the collective benefits of grazing strategies such as silvopasture or agroforestry, which contribute more than only capturing carbon.

### Meat processing—a final step in producing nutritious food

The meat processing sector completes the lifecycle by producing high-value nutrient nutrient-dense human food in addition to valuable co-products. Interaction between the grazing, intensive feeding, and processing sectors is central to maintaining consistent supply and packing plant process efficiency (Horwood et.al 2023). This sector is also the contact point for retailer Scope 3 emissions reporting, with major retailers demanding substantial reductions by 2030. Meat processing requires significant energy and water use driving an acute focus on efficiency and recycling. Table 4, displaying Australian industry data from 2008 to 2022, reflects progress, with significant recent and current progression in “behind the switch” plant actions including a strong focus on renewable energy with heat pumps, solar PV with batteries, biogas boilers and biogas capture and reuse technologies being adopted at scale (Horwood, 2023). An Australian 2022 survey data reported a 3-fold variation between the best and lower performers confirming the potential for rapid further improvement. Externally decarbonization of grid electricity will deliver 40% to 90% reduction dependent on location (Horwood and Kember, 2023).

**Table 4.** Australian abattoir energy and carbon emissions intensity by period

Resource	2008/9	2013/14	2019/20	2021/22
**Energy intensity (MJ/tHSCW)**	**4,108**	**3,005**	**3,316**	**3,435**
Carbon emissions intensity* (kgCO_2_e/tHSCW)	**554**	**432**	**397**	**447**

**Figure 5.**
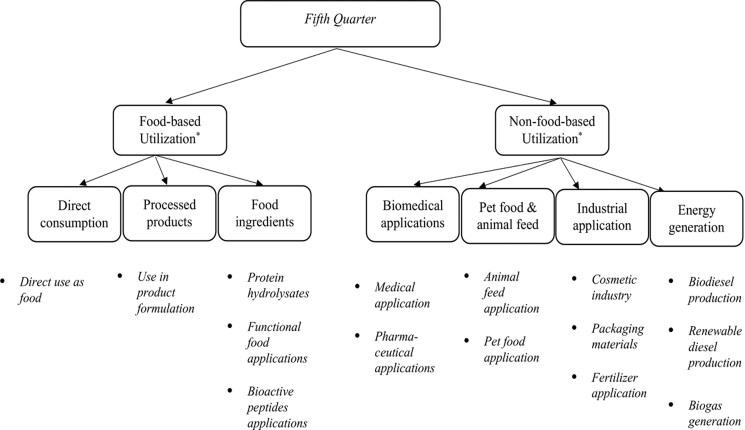
“5th quarter” utilization across industries (Soladoye et al., 2021).

In addition to sales of muscle meat and trim, co-products contribute substantial value and are critical to eliminating industry waste. While emissions metrics often relate to carcass weight this is little more than a third of the live animal, with the “5th quarter”, illustrated in Figure 5 contributing human food and environmental benefits. Offal products directly provide nutrient-dense human nutrition while other by-products provide extensive value ranging from biomedical and pharmaceutical products to fertilizer (Soladoye et.al, 2021; Coleby, 2023).

### Adaptation, disease, management, and welfare

In the context of the varying production systems and the need to adapt to locality, animal breed and adaptation is an additional consideration of the optimal livestock systems. This refers to the prevalence of internal and external parasites and diseases, associated with specific climatic conditions, as well as inherent breed tolerances to these risks. For example, the Nguni cattle of Southern Africa are well-adapted to hot, dry climates and resist parasites and diseases, making them ideal for semi-arid grazing (Kooverjee et al., 2022). The high-yielding Holstein-Friesian breed thrives in temperate climates but needs intensive management and supplemental feeding (Laible et al., 2021). In Brazil’s tropical regions, the Nelore breed is favored for its efficient feed conversion and resistance to heat and parasites like ticks (de Carvalho Porto Barbosa et al., 2023). In humid areas, livestock face diseases like bovine babesiosis, requiring regular veterinary care and strategic grazing. In temperate zones, issues such as liver fluke and respiratory diseases are more common, necessitating specific antiparasitic treatments and winter housing strategies. By selecting breeds that are well-adapted to local conditions and employing appropriate management practices, farmers can enhance resilience and productivity, contributing to sustainable agricultural systems.

Advanced management practices, such as those utilized in the United States and highlighted in Supplementary Material (S5), play a significant role in enhancing both animal performance and resilience, including via breeding adaptation. These include the use of genetic data, such as estimated progeny differences and ultrasound technology for bull selection, alongside comprehensive herd health and vaccination programs. In addition, data management is increasingly important to ensure that only the most productive and resilient animals remain in a herd, feed stocks are balanced, and animal performance is closely monitored alongside genetic improvement and optimized resource use. By integrating these practices, farmers can make informed management decisions that lower production costs, increase profitability, and reduce emissions, ultimately fostering sustainable agricultural systems that are better equipped to withstand climatic variability.

Adequate management strategies, adapted to context and breed, will in most cases also contribute to animal-welfare as it can reduce the physiological stress of the animal and hence contribute to the reduction of emissions (via lowered respiratory rate, better productivity, and reduced methane emissions). However, productivity alone does not guarantee welfare; stressors like group separation or restricted natural behaviors during lambing can harm animal well-being and reduce system success. Of course, a wider view of the pros and cons of grazing and housing for animal welfare (both from a health and social perspective) need also to be considered as a critical component of sustainability (Rivero and Lee, 2022). Therefore, factors beyond climate, such as management practices, must be considered when aiming to reduce livestock emissions. Such practices not only improve animal productivity and welfare but also contribute to more resilient ranches and ecosystems.

### Product quality and consumer preference

An additional consideration, regardless of the impact on the environment and ecosystem, is the impact that each livestock system has on both product quality and affordability. In low-income countries, many consumers are concerned with the affordability of products and thus may favor a grain-finished meat product which is generally more affordable due to the shorter finishing time of the animal and economies of scale. Conversely, in higher-income countries, consumers may be more aware of credence values i.e., non-measurable or “trust” values, such as the conditions under which the animal was raised, and are willing to pay more for labels such as free-range or pasture-fed (Henchion et al., 2014). In addition, nutritional difference also arises such as pasture-fed beef typically containing higher levels of omega-3 fatty acids, long-chain polyunsaturated fat, trans-fat, conjugated linoleic acid, and antioxidants such as vitamin E, whilst containing lower total lipids (Lukic et al., 2021; Van Vliet et al., 2021). Similar fatty acid profile differences are observed within pasture-fed dairy systems and varying intensities of supplementary feeding (Dunshea et al., 2008). Grain-fed beef, however, may contain more cholesterol-lowering fatty acids, yet these differences require further exploration (Nogoy et al., 2022). Furthermore, the recent focus on nutritional parameters as part of environmental studies may influence the emissions per nutritional unit, as opposed to per kilogram of end-product (Wilkinson et al., 2020; McNicol et al., 2024).

## Conclusion

The case studies presented from different continents demonstrate that livestock can enhance environmental outcomes while increasing the productivity of both soils and livestock. Examples include managing highly productive grasslands in Ireland, implementing complementary grazing and feedlots to protect fragile soils in low rainfall regions of Australia, and using a combination of grazing and intensive feeding in the United States to counter extreme winter conditions and boost productivity. These cases highlight the necessity of actively adapting management systems to local environments and conditions.

The case studies also illustrate that in each case, data collection and contextualization in life cycle analysis is essential to optimize the many complex system parameters. One-size-fits-all approaches do not apply, localized innovation by farmers and industry must take the lead. To meet the nutritional needs of a projected 10 billion people, livestock production efficiency must be accelerated, considering the entire life cycle from birth to plate. This involves adapting to climatic variations, from droughts to extreme winters, and efficiently balancing nutrient supply by utilizing human-inedible biomass from non-arable land and fully exploiting food by-products and waste streams. Managing non-arable grasslands and forests as productive yet eco-sensitive land, alongside optimizing crop production, is crucial.

Sustainable grazing practices such as silvopasture and agroforestry have emerged as key strategies, offering ecological benefits like enhanced biodiversity, improved soil health, and increased carbon flux fixation (below and above ground). These systems contribute to climate change mitigation and support sustainable agricultural production by creating resilient ecosystems. However, to achieve significant reductions in greenhouse gas emissions and maximize biosystem services, these practices may need to be combined with intensive feeding systems like feedlots. Feedlots control feed intake, improve livestock efficiency, and reduce the overall impact footprint of meat production. By integrating different production strategies, comprehensive approaches can be developed to address carbon flux fixation, emission reduction, soil health improvement, and agricultural intensification.

This can be done more effectively and localized further by the incorporation of GIS monitoring tools to monitor carbon stocks, vegetation, and climate anomalies, all assisting in monitoring the health and progress of each ecoregion individually. Future policies and practices must thus be adaptive and region-specific, leveraging optimal land-use practices as well as existing monitoring tools and technologies to achieve the best environmental and economic outcomes. Policies must also give room to localized innovation. Advanced monitoring and assessment tools will be essential in guiding these efforts, ensuring that sustainable livestock production can meet the growing global food demand while minimizing environmental impacts.

## Supplementary Data

Supplementary data are available at *Animal Frontiers* online.

vfae050_suppl_Supplementary_Figures_S1-S5

## Author Biography

**Mrs. Enrike Maree**

**Mrs. Enrike Maree** is a Researcher and Communications Manager at GOALSciences. She holds an MScAgric in Animal Science from the University of Pretoria with a focus on sustainability indices for the agricultural industry and is a PhD candidate at the same institution, with a focus on the sustainability of meat production from a grazing and ecological perspective. Her previous experience includes roles as a nutrition intern and a technical advisor at a leading feed company. In her current role, she conducts research aimed at enhancing sustainability in livestock production and the broader agricultural sector. Additionally, she engages in consumer education through AgreeToThisAgri. Her research interests encompass livestock sustainability, food security, sustainable environments, and the socioeconomic impacts of sustainable agriculture, with a vision of promoting a holistic, sustainable food system. **Corresponding author**: Enrike.maree@goalsciences.org

**Pablo Manzano**

**Pablo Manzano** works as an Ikerbasque Fellow at the Basque Centre for Climate Change in Bilbao, Spain. A rangeland ecologist, he graduated from the Autonomous University of Madrid and has gathered research experience at the FSU Jena, Germany, and the University of Helsinki, Finland, plus shorter stays at Landcare Research, Christchurch, New Zealand, and Texas State University, San Marcos, USA. In parallel, he has developed a career in international development cooperation, with experience at several NGOs as well as international organizations such as IUCN, FAO, and the World Bank. His experience has allowed him to perform transdisciplinary research on livestock-based socio-ecosystems.

**Terry McCosker**

**Terry** founded RCS in 1985 where he remains a Director and is Executive Chairman of Carbon Link, which he founded in 2007. He has worked in research, property management, and both public and private extension. He is a Churchill Fellow and a Fellow of the Tropical Grasslands Society, is an Honorary Doctor of Agribusiness (CQU), and was awarded an Order of Australia Medal for his contribution to agriculture. Terry is a pioneer in the fields of rangeland animal production, grazing management, holistic farmer education, and more recently soil carbon measurement and carbon farming, having been committed to research and commercial activity in this area since the early 2000’s. One of his greatest lifetime achievements has been to effectively bridge the gap between the contesting paradigms of traditional agriculture and regenerative agriculture, helping to ensure the long-term survival of Australian agriculture and its farming families.

**Michael R. F. Lee**

**Professor Michael Lee** FRSA FRSB FRASE ARAgS is an expert in sustainable livestock systems, defining their role in securing global food security at the same time as protecting environmental health (Livestock’s role in human and planetary health). He is Deputy Vice Chancellor of Harper Adams University, England’s Premier specialist agriculture and land use University. He was awarded the Sir John Hammond Memorial Prize in 2015 for services to Animal Science and fellowships of the Royal Societies of the Arts and Biology in 2019 and 2023, respectively. He was also awarded an Associateship of the Royal Agricultural Societies in 2023 and a Fellowship of the Royal Agricultural Society of England in 2024. He served as President of the British Society of Animal Science from 2018-2022 and was a Trustee of the society for 12 years. In August 2019, he was elected President of the European Federation of Animal Science Livestock Farming Systems Commission. He represents the UK on Animal Task Force, a Public-Private think-tank informing the EU parliament in Brussels, and is co-chair of the UK Universities Climate Network—Net Zero group. In 2023 he was selected to join a Technical Advisory Group of the UN’s Food and Agriculture Organization (FAO). Prof Lee also sits on the BBC’s Rural Affairs Committee, the Agriculture Advisory Group convened by the UK’s Climate Change Committee (CCC), and the Scientific Council of the World Farmers Organization (WFO).

**John Gilliland**

A special advisor to the UK’s Agriculture Horticulture Development Board (AHDB) and to Quality Meat Scotland (QMS), **John Gilliland**, is a willow and livestock farmer from N. Ireland, whose farm has independently been verified to be “Beyond” Net Zero, today. He is also the Professor of Practice in Agriculture and Sustainability at Queens University Belfast (QUB); Chair of Harper Adams University’s Sustainable Farm Networks (SFN); and chair of the innovative, EIP-Agri funded, farmer-led, carbon farming project, ARC Zero, Home | ARCZero (arczeroni.org). John chaired the writing of the N. Ireland Sustainable Land Management Strategy which led to the creation of N. Ireland’s Soil Nutrient Health and LiDAR Scheme, which is a World first and an investment of £38m to baseline all fields’ soil, trees, and hedges in N. Ireland, Soil Nutrient Health Scheme | Agri-Food and Biosciences Institute (afbini.gov.uk).

Previously, John was President of the Ulster Farmers Union, a Non-Executive Director of SRUC, Chair of DEFRA’s Rural Climate Change Forum, Vice Chair of the UK’s Sustainable Development Commission, and a member of the EU Commission’s Soil Mission Board.

**Wayne Truter**

**Prof Wayne Truter** has a PhD in Integrated Agricultural and Environmental Science from the University of Pretoria. He is currently employed by the University of the Free State as a Full Research Professor in the Faculty of Natural and Agricultural Sciences and as the Executive Manager of the Green Futures Hub. He serves as the Chairman of the Executive Board and Scientific Committee of the FPLRI Research, Technology and Innovation Institute. He is also the Research Programme Director of AF4RICA Lab, Enterprises University of Pretoria. Wayne’s professional experience entails land (*Degraded or mined agricultural land*) regeneration planning and management, with particular emphasis on understanding the interrelationships of soil, grasses, legumes, water and livestock, and wildlife. His specialization focuses on establishing the most sustainable, economical, and practically feasible regenerative land use management system in disturbed environments. This includes forage, pasture, and grazing management as an important component of a fodder flow program. He has spent 25 years conducting research and evaluating forage, pasture, and livestock production in land and community regeneration programmes and rehabilitated land management systems. He is also registered as a Professional Scientist in agricultural and environmental sciences at the South African Council for Natural Scientific Professions (SACNASP) Reg. no. 100023/01. Wayne is the founding member and 1^st^ president of LaRSSA (Land Rehabilitation Society of Southern Africa), and an Agribusiness Coach who consults nationally and internationally on land regeneration, rehabilitation, and reclamation.

**James Blignaut**

**James Blignaut** is a professor extraordinaire attached to the SPL of Stellenbosch University and an honorary research associate of SAEON. He is also the director of ASSET Research and Beatus. He is an environmental resource economist specializing in the economic costs and benefits of restoration, which includes, among others, conservation and regenerative agriculture.

**Ben Weinheimer**

**Ben Weinheimer** is president & CEO of the Texas Cattle Feeders Association (TCFA), headquartered in Amarillo, Texas. He was named to that position in 2022 and has been on staff since 1995, serving previously as vice president from 2006 to 2021 and as regulatory manager from 1995 to 2005. As president & CEO, Weinheimer is responsible for the day-to-day operations of the Association, including supervising the implementation of policy at the state and federal legislative and regulatory levels, as well as the association’s programs and services offered to members in Texas, Oklahoma, and New Mexico. Weinheimer is active in several industry-related and civic organizations and currently serves as Chairman of the Panhandle Regional Water Planning Group, member of the Llano Estacado Regional Water Planning Group, past chairman of the U.S. Roundtable for Sustainable Beef, past president of the Texas Agricultural Lifetime Leadership Alumni Association, past president of the Texas Section of the American Society of Agricultural Engineers, member of the National Cattlemen’s Beef Association, member of the Texas Association of Community Colleges Business Advisory Council, and member of the Texas A&M University—Veterinarian, Education, Research and Outreach Advisory Council. Weinheimer grew up on a diversified crop and livestock operation in the Texas Panhandle. He is an honors graduate of Texas A&M University with a degree in agricultural engineering and is a licensed professional engineer in Texas, New Mexico, and Oklahoma. Ben and wife, Jennifer, reside in Amarillo, Texas. They are proud parents of a daughter and two sons and grandparents of five amazing grandchildren.

**Lindeque du Toit**

**Dr Lindeque du Toit** is a senior lecturer in the Department of Animal Science in the Faculty of Natural and Agricultural Sciences at the University of Pretoria. He obtained his BSc(Agric) in Animal and Pasture Science and MSc(Agric) in Animal Nutrition between 1996 and 2006, and a PhD in Animal Nutrition from UP in 2017.

His research area is focused on meeting the challenge to increase the production of quality livestock protein and fiber to meet increasing demand, adapt to environmental changes, and reduce the impact of livestock production systems on the environment. His current research projects are focused on addressing these challenges by firstly developing and improving country-specific greenhouse gas emission factors for all classes of livestock in South Africa including privately owned game and secondly by identifying and evaluating practical mitigation strategies that have a high probability of being adopted by livestock producers and industry.

Dr Du Toit lectures subjects in livestock production systems, pasture and range management, animal nutrition, and small stock nutrition and management. He is currently a serving member of the South African Society for Animal Science and a member of the South African Council for Natural Scientific Professionals.

**Rod Polkinghorne**

**Rod Polkinghorne** is a highly experienced research team leader who has pioneered the use of untrained consumer sensory evaluation as a primary tool to quantify the eating quality impact of multiple production factors from genomics through farm management, transport, processing, maturation, value-adding, and cooking method on individual beef and lamb muscles. This data, from 11 countries and 200,000 consumers, have been used to build the Meat Standards Australia and other consumer prediction models which underpin grading at a cut level and support strong retail branding. Rod is an adjunct professor at the University of New England and was awarded an honorary doctorate from Murdoch University and an Order of Australia in recognition of services to the beef industry. He has been directly involved in feedlotting, grassland breeding and fattening, beef fabrication, and retailing with this strong commercial background assisting in driving the application of scientific outcomes at farm, factory, retail, and government level. His work has included strategic industry evaluation and engagement in advancing and advocating for evidence-based decision-making relating to animal agriculture’s essential role in human nutrition, and environmental and societal structures.
