# Supplementary material for: Ruminant livestock farmers and industry are leading innovation to deliver human nutrition and improved environmental outcomes through sector lifecycle collaboration: a review of case studies
Source: Anim Front. 2025 Apr 5;15(1):55–71. doi: 10.1093/af/vfae050 (PMC11971517; doi:10.1093/af/vfae050)
Supplement: vfae050_suppl_Supplementary_Figures_S1-S5 [file vfae050_suppl_supplementary_figures_s1-s5.docx]

**Supplementary Material**

**S1. Australia**

**1.1 Overview and Aim**

This case study evaluates the outcomes of large-scale soil carbon sequestration projects in Queensland and New South Wales, Australia. The aim is to assess the effectiveness of these projects in enhancing soil carbon stocks, reducing greenhouse gas emissions, and generating Australian Carbon Credit Units (ACCUs)

**1.2. Study Site Description** The study was conducted across various properties in Queensland and New South Wales, Australia. These properties, encompassing a total area of approximately 18756 hectares, are characterized by low rainfall and fragile soils unsuitable for cropping. The land is primarily utilized for grazing cattle and is part of large-scale soil carbon projects registered with the Australian Government. The properties feature diverse landscapes, including areas with black cracking clays and shallow traprock soils.

**1.3. Study Description and Design**
The soil carbon projects were baselined in 2016 by Carbon Link under the 2014 method, remeasured in 2021 using the 2018 method, and credited in 2023 under the 2021 method. Intact soil cores were taken to a depth of 120 cm and analyzed using LECO in 2016 and SCANS technology by CSIRO in 2021. The practice change involved intensification of grazing to enhance carbon sequestration. The project areas ranged from 578 to 4,352 hectares, with measurements made every 5 cm down the core and reporting based on equivalent soil mass.

Incorporating a Life Cycle Assessment (LCA) approach, the study carefully captured and analyzed data related to soil carbon changes, emissions from livestock and fuel, and the overall environmental impact. This approach provided a comprehensive understanding of the project's outcomes, enabling more informed decision-making regarding the optimization of grazing practices for both environmental and economic benefits.

**1.4. Outcomes**

- **Soil Carbon Sequestration**: The total drawdown across the four properties was 467,175 t CO2e on the project area, significantly higher than the total emissions of 26,355 t CO2e from livestock and fuel. This indicates a net carbon sequestration effect, with beef produced on these properties having a carbon-negative footprint, drawing down 32 to 61 t CO2e for each tonne of livestock carried over five years.
- **Carbon Credits Issued**: The projects generated 258,808 t CO2e in credits after statutory discounts, with ACCUs issued based on the increase in soil carbon stocks.
- **Soil Carbon Distribution**: A substantial portion of carbon sequestration occurred below 30 cm, with properties B to E sequestering 21% to 69% of carbon at these depths. This deep soil carbon storage highlights the potential for long-term carbon sequestration.
- **Water Holding Capacity**: Increased soil organic carbon improved water holding capacity, estimated at 247,000 to 279,000 m³ for the properties. This enhancement in water use efficiency contributed to better pasture productivity and resilience to drought.
- **Emission Intensity and Economic Impact**: The emission intensity per hectare varied, with net emission intensities indicating carbon-negative production. The carbon EBIT ranged from -$4 to $165 per hectare per year, while livestock EBIT varied due to differences in stock management and drought conditions.

**1.5. Conclusions**
The large-scale soil carbon projects in Australia demonstrated significant potential for carbon sequestration through intensive grazing management. The integration of data capture and Life Cycle Assessment (LCA) was crucial in providing a holistic understanding of the environmental and economic impacts. This approach enabled the optimization of practices, leading to substantial net carbon sequestration, improved soil health, and increased water retention. The generation of ACCUs provided an economic incentive for farmers to adopt sustainable practices, ensuring both environmental and economic sustainability.

**S2. Northern Ireland**

**2.1 Overview and Aim**

The ARC Zero project in Ireland is a pioneering initiative aimed at demonstrating the potential for farms to achieve net-zero carbon emissions through innovative management practices and carbon sequestration strategies. The project, which began in 2021, seeks to provide high-quality, science-based evidence to counter criticisms of the farming sector's environmental impact and to showcase the ability of farms to capture, manage, and store carbon effectively.

**2.2 Study Site Description**The ARC Zero project involves seven farms across Northern Ireland, each representing different farming types, including dairy, beef, sheep, and arable systems. These farms are located in various regions, including Armagh, Down, Antrim, Londonderry, and Fermanagh. The diversity of these farms provides a comprehensive overview of the different challenges and opportunities for carbon management in Irish agriculture.

**2.3 Study Description and Design**The project was initiated by establishing a baseline for carbon stocks through detailed soil sampling, analysis, and aerial scanning via LiDAR. Each farm was divided into 2-hectare blocks, and core soil nutrient samples were taken to a depth of 7.5 cm in grassland and 15 cm in arable fields. The analysis included measurements of soil pH, phosphate, potassium, calcium, magnesium, and organic matter. Below carbon measurements were taken to a minimum of 30cm, with Soil Organic Carbon and bulk density sampling done at 0-15cm, 15- 30cm, 30cm to 60cm and 60cm to 100cm. Above-ground carbon was measured in trees and hedgerows using aircraft-mounted LiDAR to form precise 3D images.

Comprehensive audits of greenhouse gas (GHG) emissions were conducted using the SAC’s accredited AgreCalc tool. Data on stock numbers, animal production performance, feed use, fuel, and fertilizer use, as well as fodder conservation and electricity consumption, were captured. A Life Cycle Assessment (LCA) approach was employed to analyze the environmental impacts across the entire farming system, providing a detailed understanding of carbon flows and opportunities for reducing emissions.

**2.4 Outcomes**The initial phase of the project revealed that farmers manage substantial carbon stocks, with a total of over 515,000 tonnes of CO2e stored across the seven farms. Two farms were found to be already beyond net-zero emissions, capturing more carbon and displacing fossil fuels, than they emit. The second phase of the project focused on practical steps to further reduce GHG emissions and increase carbon capture, including the use of multispecies swards, strategic tree planting, and improved animal health and genetics.

Key findings, based on the premise, if you cannot measure, you cannot manage, include significant reductions in carbon emissions across all participating farms, with some achieving reductions of up to 53% in CO2e per kilogram of product. The use of LiDAR provided valuable insights into field features, aiding in the placement of buffer strips and the management of erosion-prone areas to improve water quality, biodiversity as well as increase carbon stocks.

**2.5 Conclusions**The ARC Zero project demonstrates that where farmers know their precise environmental numbers, this knowledge allows them to target management practices and with comprehensive monitoring, farms can significantly reduce their carbon footprint, contribute to climate change mitigation and deliver wider environmental goods. The project's success highlights the importance of high-quality data and robust measurement tools in achieving net-zero emissions. The findings underscore the decision by the Northern Ireland Government for broader application of these practices across the agricultural sector, suggesting all farmers could achieve similar results through their new £38m, Soil Nutrient Health Scheme. This project serves as a model for integrating sustainable farming practices with advanced carbon management techniques, proving that agriculture can play a crucial role in achieving climate and wider environmental goals. The project further show that precise data capture and a Life Cycle Assessment (LCA) approach are essential for effective environmental management in agriculture. By leveraging these tools, farms can significantly reduce their carbon footprint and contribute to climate change mitigation. The success of this project highlights the critical role of comprehensive monitoring and analysis in achieving net-zero emissions and broader environmental goals.

**2.6 Report links:** [Arc-Zero-Final-Report.pdf (cafre.ac.uk)](https://www.cafre.ac.uk/wp-content/uploads/2023/10/Arc-Zero-Final-Report.pdf) ; [Soil Nutrient Health Scheme Zone 3 uptake | Agri-Food and Biosciences Institute (afbini.gov.uk)](https://www.afbini.gov.uk/news/soil-nutrient-health-scheme-zone-3-uptake)

### **S3. Feedlots**

**3.1 Overview and Aim**

This case study evaluates the role of feedlots in Australia’s beef industry, focusing on their historical role in drought-proofing, maintaining consistent year-round beef supply, and enhancing beef quality. It assesses the environmental impact of feedlots and their efficiency in terms of feed, growth rates, and manure utilization.

**3.2 Study Site Description**

The study includes five feedlots across Queensland, New South Wales, and Victoria, covering a range of climates and operational scales. These feedlots have capacities ranging from 3,000 to 30,000 head, with annual turnoffs between 8,000 to 90,000 head. The feedlots are representative of the industry, reflecting both regional commodity production and market destinations.

**3.3 Study Description and Design**

The feedlots were surveyed in 2023 to collect data on capacity, turnoff, feed composition, and manure management. The feed used in rations includes by-products like whole cottonseed and roughage harvested locally, with grain grades unsuitable for human consumption. Manure management practices include using liquid effluent for crop irrigation and composting solid manure for agricultural use. A Life Cycle Assessment (LCA) approach was incorporated to capture data on feed efficiency, emissions from manure, and the overall environmental impact of the feedlot operations. This approach provided a detailed analysis of the entire feedlot system, enabling a better understanding of its sustainability and areas for improvement.

**3.4 Outcomes**

- **Feed Efficiency and Composition:** Feed efficiency is enhanced through growth rates, with rations primarily consisting of by-products and lower-grade grains unsuitable for human consumption. This reduces competition for human food resources.
- **Manure Utilization:** Liquid effluent is used to irrigate crops or grassland, while solid manure is composted or spread on cropland. Manure utilization provides critical nutrients and benefits soil structure, health, and water holding capacity.
- **Environmental Impact:** Feedlots help in drought-proofing by moving cattle from pastures to feedlots during dry periods, preventing overgrazing and pasture damage. The complementary nature of grass and feedlot sectors facilitates efficient pasture and animal growth across variable seasonal conditions.

**3.5 Conclusions**

Australian feedlots play a crucial role in the beef industry by ensuring a consistent year-round supply of high-quality beef. The integration of data capture and Life Cycle Assessment (LCA) in this study provided critical insights into the feedlot's environmental impacts and areas for optimization. Future efforts should focus on refining feed compositions, improving manure management techniques, and further integrating feedlot operations with broader environmental and sustainability goals.

### **S4. South Africa**

**4.1 Overview and Aim**

This case study assesses the impact of ultra-high-density grazing (UHDG) on veld condition in the Free State, South Africa. The aim is to evaluate the effectiveness of UHDG in improving veld condition, grass species diversity, and biomass production compared to selective grazing and no-grazing control sites.

**4.2 Study Site Description**

The study was conducted southeast of Reitz in the Free State province. The farm features undulating topography with diverse soil types, primarily used for grazing. UHDG was implemented in December 2017, involving hourly movement of a herd of approximately 500 Drakensberger cows within electrified strips during summer and the use of crop residues during winter.

**4.3 Study Description and Design**

Veld condition surveys were conducted at seven sites in February 2019 and February 2021. Five sites practiced UHDG, while two control sites practiced selective grazing, and one site had no grazing. Data collected included grass species composition, biomass production, veld condition score (VCS), and grazing capacity. Grass biomass was measured using a disc pasture meter, and species composition was recorded along 100 m transects using the step-point method. To provide a thorough analysis of the environmental impacts and benefits of UHDG, a Life Cycle Assessment (LCA) approach was applied. This involved capturing data on soil health, carbon sequestration, and biodiversity improvements, enabling a comprehensive understanding of the sustainability of UHDG practices.

**4.4 Outcomes**

- **Grass Species Composition:** UHDG sites showed an increase in decreaser grasses from 30.9% to 32.1%, compared to 15% at selective grazing sites and 0% at no-grazing sites. Increaser grasses, indicative of disturbed or overgrazed conditions, decreased at UHDG sites.
- **Veld Condition Score (VCS):** The average VCS at UHDG sites increased from 37.5% in 2019 to 45.4% in 2021. Selective grazing sites had a VCS of 30%, while no-grazing sites had a VCS of 21.6%.
- **Biomass Production:** UHDG sites produced an average of 5,212 kg/ha of dry grass, compared to 3,153 kg/ha at selective grazing sites and 6,760 kg/ha at no-grazing sites. However, biomass at no-grazing sites included a high proportion of moribund material.
- **Grazing Capacity:** Based on biomass, UHDG sites had a grazing capacity of 2.7 ha/LSU, while selective grazing sites had 5 ha/LSU, and no-grazing sites had 3 ha/LSU.
- **Grass Species Diversity:** UHDG sites increased from 17 species in 2019 to 23 in 2021. Selective grazing sites remained constant at 11 species, while no-grazing sites decreased from 17 to 9 species.

**4.5 Conclusions**

UHDG demonstrated significant improvements in veld condition, grass species diversity, and biomass production compared to selective grazing and no-grazing sites. The use of data capture and a Life Cycle Assessment (LCA) was essential in understanding these impacts and providing evidence for the sustainability of UHDG practices. These positive changes suggest that UHDG is a sustainable grazing management approach. However, ongoing monitoring and expansion of the study to include additional ecological parameters are recommended to fully understand the long-term impacts of UHDG.

### **S5. GIS monitoring tools.**

Effective monitoring of rangelands and ecosystem biodiversity is essential for sustainable management and conservation. Various advanced technologies provide detailed insights into the health and productivity of these ecosystems. Here, we discuss several key tools and their applications:

#### **1. SATNAV (Satellite Navigation)**

SATNAV systems, like GPS, are fundamental for precise geolocation and mapping. They help track changes in vegetation cover, monitor animal movements, and manage land resources efficiently. The integration of SATNAV with other remote sensing technologies enhances the accuracy and reliability of ecological data collection.

#### **2. Near-Infrared Spectroscopy (NIR)**

NIR technology is used for analyzing soil and plant properties. It measures the reflectance of near-infrared light, which is sensitive to organic matter, moisture content, and nutrient levels in soil and vegetation. NIR can be deployed through handheld devices or integrated into drones and satellites for large-scale assessments.

#### **3. Light Detection and Ranging (LIDAR)**

LIDAR technology uses laser pulses to create high-resolution, three-dimensional maps of the land surface. It is particularly useful for measuring vegetation structure, biomass, and topography. LIDAR can penetrate forest canopies to assess understory vegetation and ground surface, making it a valuable tool for comprehensive ecosystem monitoring.

#### **4. Synthetic Aperture Radar (SAR)**

SAR employs microwave radar to create detailed images of the Earth's surface. It is effective in all weather conditions and can penetrate clouds and vegetation. SAR is used to monitor soil moisture, surface roughness, and land deformation, providing critical data for understanding ecosystem dynamics and managing rangelands.

#### **5. Aerial Imagery**

High-resolution aerial imagery, captured by drones or aircraft, offers detailed visual information about the landscape. These images help in mapping vegetation types, detecting invasive species, and assessing land use changes. The high spatial resolution makes it ideal for localized studies and precise management interventions.

#### **6. Satellite Imagery**

Satellite imagery varies in resolution and applications:

- **High Resolution**: Satellites like WorldView and QuickBird provide detailed images (less than 1 meter resolution) suitable for fine-scale studies, such as urban planning and detailed vegetation mapping.
- **Moderate Resolution**: Satellites like Landsat and Sentinel-2 offer images with resolutions of 10-30 meters. They are widely used for regional land cover mapping, vegetation health monitoring, and agricultural assessments.
- **Coarse Resolution**: Satellites like MODIS provide images with resolutions of 250 meters to 1 kilometer. These are useful for large-scale ecological studies, climate change research, and tracking global vegetation dynamics.

### **Monitoring Methane, Carbon Stock, and Greenhouse Gas Emissions**

Effective management of rangelands also involves monitoring methane emissions, carbon stock, and overall greenhouse gas (GHG) emissions. Various tools and technologies are employed for these purposes:

#### **1. Methane Emissions Monitoring**

Methane, a potent greenhouse gas, is a significant concern in rangeland management, especially in livestock farming. Technologies used include:

- **Infrared Gas Analyzers**: Measure methane concentrations in the atmosphere by detecting infrared absorption.
- **Open-Path Laser Systems**: Provide real-time measurements of methane emissions over large areas.
- **Remote Sensing**: Satellites and aircraft equipped with spectrometers can detect and quantify methane plumes.

#### **2. Carbon Stock Monitoring**

Monitoring soil and vegetation carbon stocks is crucial for understanding carbon sequestration and emissions. Key tools include:

- **Soil Sampling and Laboratory Analysis**: Traditional method for measuring soil organic carbon.
- **Portable X-ray Fluorescence (pXRF)**: Allows rapid, in-field analysis of soil properties.
- **Remote Sensing**: NIR, LIDAR, and multispectral imaging help estimate vegetation biomass and soil carbon content.
- **Automated Soil Respiration Chambers**: Measure CO2 flux from soil, providing data on soil carbon dynamics.

#### **3. Greenhouse Gas Emissions Monitoring**

Monitoring overall GHG emissions involves a combination of ground-based and remote sensing techniques:

- **Eddy Covariance Systems**: Measure fluxes of CO2, methane, and other gases between the land surface and the atmosphere.
- **Flux Towers**: Equipped with various sensors to monitor GHG emissions continuously.
- **Spectroscopic Gas Analyzers**: Provide precise measurements of multiple GHGs in the atmosphere.

### **Integrated Monitoring Approaches**

Combining multiple tools and data sources enhances the accuracy and comprehensiveness of ecosystem monitoring. Geographic Information Systems (GIS) integrate spatial data from various sensors, providing a platform for analysis and visualization. Advanced machine learning algorithms are increasingly used to analyze complex datasets, identify patterns, and predict ecological changes.

### **Conclusion**

The integration of advanced monitoring tools is essential for sustainable rangeland management and biodiversity conservation. By leveraging technologies like SATNAV, NIR, LIDAR, SAR, and various satellite imagery resolutions, we can obtain detailed insights into ecosystem health. Additionally, specialized tools for monitoring methane, carbon stock, and GHG emissions enable effective management strategies to mitigate climate change impacts and enhance ecological resilience. These integrated approaches ensure that rangeland and ecosystem management practices are data-driven, adaptive, and capable of supporting sustainable development goals.
